# New Cure for Intermittent Fever

**Published:** 1857-08

**Authors:** 


*New Cure for Intermittent Fever.*—R. H. Hadsden, M. D., in a report to the semi-annual session of the *East Tennessee Medical Society*, recommends the use of *prepared* chalk and vinegar for intermittent fever. He says: "If the remedy should succeed in other hands as it has with me, it will prove a valuable acquisition in Intermittent Fever, especially the chronic form of that disease, though it may never supercede quinine in the cure of cases of recent occurrence."—*Southern Journal of Medical and Physical Science.*

